# Protein kinase C showcases allosteric control: activation of LRRK1

**DOI:** 10.1042/BCJ20220507

**Published:** 2023-02-10

**Authors:** Hannah Tovell, Alexandra C. Newton

**Affiliations:** Department of Pharmacology, University of California, San Diego, La Jolla, CA 92093, U.S.A.

## Abstract

Allosteric regulation of multi-domain protein kinases provides a common mechanism to acutely control kinase activity. Protein kinase C serves as a paradigm for multi-domain proteins whose activity is exquisitely tuned by interdomain conformational changes that keep the enzyme off in the absence of appropriate stimuli, but unleash activity in response to second messenger binding. Allosteric regulation of protein kinase C signaling has been optimized not just for itself: Alessi and colleagues discover that protein kinase C phosphorylates LRRK1, a kinase with even more domains, at sites on its CORB GTPase domain to allosterically activate LRRK1.

## Introduction

**Figure 1.**
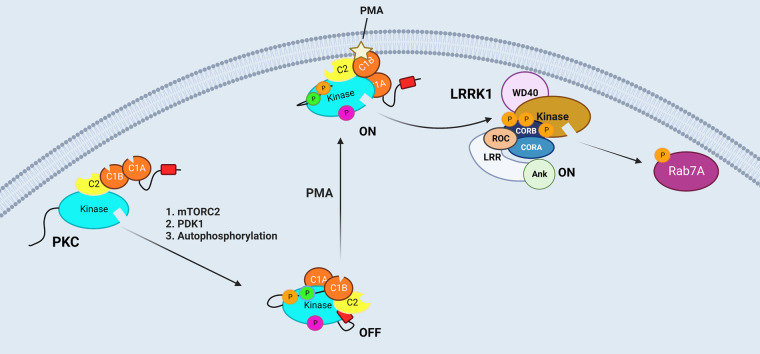
PKC is allosterically regulated by phosphorylation and lipid binding and, in turn, allosterically regulates the kinase LRRK1. Upon translation, nascent PKC is processed by phosphorylation (circles with P) at the turn motif by mTORC2, activation loop by PDK1 and the hydrophobic motif by autophosphorylation to form an autoinhibited state in the cytosol. In response to second messengers or phorbol esters such as PMA, PKC translocates to the plasma membrane and undergoes conformational changes resulting in removal of the pseudosubstrate (red rectangle) from the active site to allow substrate phosphorylation. In their new study, Malik et al. have demonstrated that PKC can phosphorylate and activate the kinase LRRK1 on its CORB domain. The authors propose that these phosphorylations stabilize an unstructured region of the CORB to allosterically activate the kinase domain. LRRK1 can then phosphorylate its downstream substrate Rab7A which is also located at the plasma membrane. Created with BioRender.com.

Protein kinase C (PKC) is no stranger to allosteric regulation. The newly synthesized protein undergoes a series of ordered phosphorylations both by other kinases (mTORC2 and PDK1) and by autophosphorylation in order for it to adopt an autoinhibited conformation [1]. This phosphorylated species is primed for activity but maintained in an autoinhibited conformation by a pseudosubstrate that binds the substrate-binding cavity, interactions of its ligand-binding C1 and C2 domains with the kinase domain, and phosphate at the autophosphorylation site. Binding of appropriate second messengers recruits PKC to the membrane, which releases the pseudosubstrate allowing substrate phosphorylation and downstream signaling (Figure 1).

Identifying bona fide substrates of PKC has been challenging given the multiplicity of isozymes (nine genes encode PKC isozymes) which have similar substrate specificities and, for the diacylglycerol-regulated conventional (PKC α, β, γ) and novel (PKC δ, ε, θ, η) isozymes, similar regulatory mechanisms. Phorbol esters, functional analogs of diacylglycerol, have proven to be powerful pharmacological tools to interrogate PKC signaling because, unlike diacylglycerol, they are not readily metabolized and thus lock PKC at the membrane in an active conformation. However, few of the plethora of phosphorylation changes resulting from phorbol ester treatment of cells have been mechanistically connected to direct phosphorylation by PKC, and functional impacts are largely uncharacterized. Yet PKC isozymes are well known to play important roles in cellular homeostasis, with aberrant activity associated with diverse diseases. Even small changes in levels of activity can be pathogenic, as epitomized by Alzheimer's Disease germline variants in PKCα that increase activity by a modest 30% yet have been shown to be sufficient to drive cognitive decline in a mouse model [2]. Understanding how PKC exerts its specific cellular functions has lagged behind the understanding of its own regulation.

In a series of beautifully executed biochemical experiments, Malik et al. have now shown that many PKC isozymes phosphorylate and activate LRRK1, but not the related isozyme LRRK2. LRRK1 and LRRK2 are multi-domain Ser/Thr kinases whose deregulation causes severe but distinct pathologies [3]. Rare germline loss-of-function mutations in LRRK1 lead to the bone disorder osteosclerotic metaphysial dysplasia and rare germline gain-of-function mutations in LRRK2 cause Parkinson's disease. Both isozymes share a number of common domains regulating protein–protein interactions and activity. Both isozymes contain ANK, LRR, ROC-CORA-CORB GTPase, kinase, and WD40 domains. LRKK2 has an additional N-terminal ARM domain. Despite significant structural similarities, the two isozymes have unique regulatory mechanisms and distinct substrates. Notably, LRRK1 is activated by EGF or phorbol ester treatment of cells [4], whereas LRRK2 is insensitive to these agonists and, instead, is regulated by recruitment to membrane vesicles via interactions of its unique ARM domain to specific Rab GTPases such as Rab29 [5–7]. Additionally, whereas both isozymes phosphorylate Rab GTPases, significant specificity exists: for example, LRRK1 specifically phosphorylates Rab7A [4] and LRRK2 specifically phosphorylates Rabs including Rab8, Rab10 and Rab12 but not Rab7A [3,8,9].

## LRRK1 is activated by PKC

Alessi and colleagues originally noted that phorbol ester stimulation of mouse embryonic fibroblasts resulted in increased phosphorylation of Rab7A at Ser72 by a mechanism that depended on the presence of the LRRK1 gene and was prevented by inhibitors of either LRRK1 or PKC [4]. In their new study, the authors show pure PKC isozymes catalyze the phosphorylation of LRRK1 *in vitro* to increase its phosphorylation of Rab7A (Figure 1). Isozymes in each PKC family were shown to phosphorylate LRRK1 *in vitro*, with PKCα, PKCβ and PKCθ exhibiting the strongest phosphorylation, whereas cellular studies were largely performed with overexpression of PKCα or activation of endogenous PKC isozymes with PMA. They map the phosphorylations to a segment in the CORB domain that is unique to LRRK1, identifying Thr1061, Ser1064, Ser1074, and Thr1075 as sites phosphorylated *in vitro* by PKC. Of these residues, Thr1075 lies within an optimal pan-PKC consensus sequence of RxRxxpTF [10]. Mutation of this residue to Ala abolished phorbol ester-induced LRRK1 activation, with mutation of Ser1064 or Ser1074 causing modest reductions in phorbol ester-induced activity (mutation of Thr1061 had no effect). Mutation of all three sites (Ser1064, Ser1074, Thr1075) to Glu modestly increased basal activity, suggesting the added negative charges may function as weak phosphomimetics. Curiously, mutation of Thr1075 to Glu abolished basal and phorbol ester-induced activity, suggesting that a single Glu at the key PKC site does not function as a phosphomimetic. This is not surprising as the carboxylate of Glu has considerably less electronegativity than a phosphate and a significantly smaller hydration sphere and volume [11]. Remarkably, phosphorylation to a stoichiometry of 0.2 mol phosphate per mol LRRK1 increased LRKK1 activity ∼100-fold, suggesting that stoichiometric phosphorylation may increase activity by three orders of magnitude especially considering a potential maximal stoichiometry of three mol phosphate per mol LRRK1. These results establish phosphorylation of Thr1075 and potentially adjacent sites as a powerful switch to turn on LRRK1 kinase activity.

**Figure 2.**
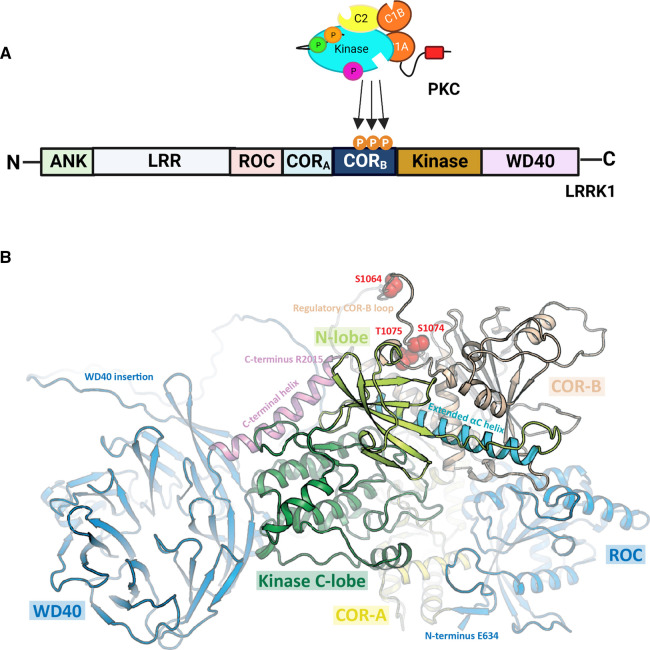
PKC phosphorylates LRRK1 on three residues in the CORB domain, altering its conformation for allosteric regulation of the kinase domain. (**A**) The study by Malik et al. demonstrates that PKC phosphorylates at least three sites, Ser1064, Ser1074, and Thr1075, which are located in the CORB domain of LRRK1. Created with BioRender.com. (**B**) Studying the AlphaFold model of LRRK1 structure, the authors identified that phosphorylation occurs in an unstructured loop region of the CORB domain and propose that phosphorylation stabilizes the CORB resulting in strengthened interactions and ordering of the kinase domain αC helix (Cyan helix in N-lobe). Figure reproduced from [3] under the creative commons license.

How does the PKC switch on LRRK1 work so effectively? Although there is a 5.8 Å cryo-EM structure of the unphosphorylated ROC-COR-kinase-WD40 segment of LRRK1, with the inactive conformation of the kinase, no structure of the phosphorylated form has been solved. However, the authors noted that the AlphaFold-predicted structure of LRRK1 has an active conformation of the kinase as indicated by a well ordered αC-helix. The orientation of this universally conserved helix defines the active vs inactive conformation of kinases, positioned ‘in’ in the active conformation and ‘out’ in the inactive conformation, stabilized by the DFG/DYG and HRD motifs of the activation loop [12]. The authors noted that the phosphorylation segment in the CORB domain was disordered and hypothesize that phosphorylation might order the region to stabilize the αC helix in the active conformation (Figure 2). In support of this idea, the comparable segment in LRRK2 (which is not regulated by phosphorylation) interacts with the αC helix to stabilize the active conformation [7]. Furthermore, inducing the active conformation of LRRK2 by addition of the Type 1 inhibitor MLi-2 was shown to stabilize the equivalent CORB residues as assessed using hydrogen-deuterium exchange mass spectrometry [13]. Taken together, the authors present a plausible model that phosphorylation of the PKC sites in the CORB domain promotes an interaction with the αC helix to stabilize the active conformation of the kinase. This previously undescribed activation mechanism adds to the repertoire of methods kinases use to stabilize the αC helix for catalysis. In the time since publication, during the writing of this commentary, a recent study disclosed two cryo-EM structures of autoinhibited LRRK1, with two inhibitory mechanisms. One mechanism required LRRK1 dimerization with the N-terminal ANK domain blocking the kinase domain from substrate binding *in trans* [14]. The structures also identified density for the unstructured CORB loop 1048–1085 to thread itself into the active site of the kinase domain as a second layer of autoinhibiton, with F1065 (+1 from S1064 PKC-mediated phosphorylation site) positioned where Y1410 of the DYG motif would normally bind the αC-helix, enforcing a ‘DYG-out' conformation. This model suggests an alternative mechanism by which, rather than inducing binding, phosphorylation of the CORB loop by PKC likely releases this autoinhibition, allowing for a ‘DYG-in' conformation and a stabilized αC-helix. In support of this second model, F1065A mutation was synergistic with Glu mutation of the three phospho-activation sites to increase basal LRRK1 activity [14].

A hallmark of PKC activation is that it occurs at the membrane, with Ca^2+^-regulated PKC isozymes translocating primarily to plasma membrane via their phosphatidylinositol-4,5,-bisphosphate (PIP_2_)-sensing C2 domain and novel PKC isozymes translocating primarily to Golgi [15]. Unsurprisingly, cellular fractionation studies revealed that Rab7A was primarily membrane-localized and, additionally, phosphorylated Rab7A was exclusively membrane-localized. A significant fraction of the cell's LRRK1 was also membrane-localized. Furthermore, immunohistochemical studies showed that phorbol esters resulted in not only the translocation of PKCα to the plasma membrane, but the accumulation of phosphorylated Rab7A. These results are consistent with PKCα phosphorylating plasma membrane-localized LRRK1, switching it on to phosphorylate membrane-bound Rab7A.

As the authors note, LRRK1 may be only the second kinase demonstrated to be directly phosphorylated and activated by PKC. Protein kinase D (PKD) is phosphorylated by novel PKC isozymes on its activation loop, a segment near the entrance to the active site that stabilizes the active conformation upon phosphorylation [16]. Thus, this phosphorylation of PKD directly regulates the kinase domain. In marked contrast, the LRRK1 regulation occurs by a unique allosteric mechanism outside the kinase domain.

As discussed above, phorbol esters such as PMA have been powerful tools to study the activity and roles of PKCs, as these non-metabolizable diacylglycerol analogs maximally and irreversibly activate PKC isozymes to reveal downstream pathways. While in this study PMA was used to activate PKC, it would be interesting in future work to determine whether natural agonists of PKC, for example activation of phospholipase C through GPCRs also resulted in the phosphorylation and activation of LRRK1. Additionally, LRRK2 phosphorylation in the switch II domain of substrate Rabs has been shown to alter Rab effector interaction and therefore downstream signaling [8,17–19], however downstream effects of Rab7A phosphorylation at Ser72 is yet to be studied. Whereas LRRK2 is known to phosphorylate a number of Rab GTPase family members, so far only Rab7A has been well characterized as a substrate of LRRK1. It will be interesting to determine in future work whether other Rabs, or perhaps other small GTPases, are additional substrates to this understudied kinase.

In summary, the work from Malik et al. identifies a unique on/off switch of the LRRK1 kinase domain that is distally controlled by CORB domain phosphorylation catalyzed by protein kinase C. This elegant allosteric mechanism exemplifies the diverse methods nature uses to exquisitely control kinase function, and underscores how a small change in structure confers regulation by the PKC pathway to LRRK1 but not LRRK2, creating regulatory specificity between these two enzymes.

## Abbreviations

PKC: Protein kinase C
PKD: Protein kinase D
